# Assessing public awareness of clubfoot and knowledge about the importance of early childhood treatment: a cross-sectional survey

**DOI:** 10.1186/s12887-019-1740-z

**Published:** 2019-10-17

**Authors:** Abdulmonem Alsiddiky, Salman Alrwibaah, Abdulrahman Alqahtani, Abdulmalek Alnujidi, Abdullah Alhomaidhi, Abdulla Almasoud, Raheef Alatassi

**Affiliations:** 1 Department of Orthopedics, College of Medicine, Research Chair of Spinal Deformities, King Saud University, Riyadh, Saudi Arabia; 2 Security Forces Hospital, Department of Orthopedic Surgery, P.O. Box: 3643, Riyadh, 11481 Saudi Arabia

**Keywords:** Clubfoot, Awareness, Perception, Foot deformity, Congenital deformity, Talipes equinovarus

## Abstract

**Background:**

Clubfoot is a treatable abnormality that can be managed with early intervention. However, there is a lack of public knowledge regarding clubfoot, which can delay treatment. This study aimed to assess the public awareness of clubfoot and knowledge regarding the importance of treatment in early childhood.

**Methods:**

This cross-sectional survey spanned 6 months, from June through November 2018, and involved persons living in Saudi Arabia. To collect data on public awareness of clubfoot risk factors, treatment, and prognosis, a questionnaire was developed by orthopedic experts and disseminated online. The target population included people of both genders and all age groups from the general population, regardless of their knowledge of someone with clubfoot.

**Results:**

By the end of the study period, 746 participants completed the online survey. In total, 520 of the respondents (69.7%) had never heard about clubfoot syndrome. Among the participants, 5.4% had a child with clubfoot syndrome and 4.6% were aware of clubfoot because they had an affected child. The top resource accessed by respondents for obtaining knowledge about clubfoot was social media channels (38.4%), followed by obtaining knowledge from relatives and friends (19.9%). The most reported perceived cause of clubfoot was hereditary and genetic disorders (58.4%), followed by neurological disorders (39.9%).

**Conclusions:**

Results show that there is low public knowledge of clubfoot which may be attributed to a lack of awareness campaigns. We recommend increasing awareness regarding clubfoot through social media platforms and public campaigns in key locations, such as malls, as this may encourage people to seek early treatment. This is important because early management of clubfoot is less invasive and with regular follow-up, leads to better patient outcomes.

## Background

Clubfoot is a congenital structural deformity characterized by hindfoot equinus, midfoot cavus, and forefoot adduction. It is the most common musculoskeletal birth defect worldwide with males being more affected than females [1, 2]. Without treatment, clubfoot may lead to lifelong disability. The affected person may not be able to wear shoes and may experience severe pain during walking [1]. Fortunately, early treatment can correct the foot position without surgery. Conversely, delayed management of the condition makes it difficult to offer non-surgical treatment [3].

Clubfoot is mainly detected clinically, and radiography is not essential for diagnosis. In addition, ultrasound can be used for antenatal diagnosis [4]. Several treatment methods are available for managing clubfoot, and these can be classified into non-operative and operative techniques. Serial manipulation and casting are non-operative treatments of clubfoot, and several methods have been described [5]. One of these is the Ponseti method, which is considered the gold standard used in most countries and is reported to have a high success rate [6]. Operative methods are used in cases of late detection or after failure of non-operative methods. In both operative and non-operative treatments, regular follow-up is mandatory to assess improvement, prevent relapse, and monitor for complications. A child with clubfoot has a risk of relapse up to 7 years of age, irrespective of the treatment method. Thus, it is important that parents are compliant with treatment and well-educated about the condition and its prognosis in order to decrease the risk of relapse.

Public knowledge and perception of clubfoot are key to early management of the condition [3], while lack of awareness is considered a barrier to treatment [1]. However, there is a paucity of studies assessing public knowledge of and perception about clubfoot worldwide, and the few studies available show low awareness in the general population regarding the condition [1–3]. This study aimed to assess public awareness of clubfoot and knowledge regarding the importance of early childhood treatment in order to identify the current gaps in public knowledge. We hypothesized that the assessment would show low levels of public awareness and knowledge for all indicators. The results of the present study will help inform a strategy for a public awareness campaign with the aim of improving rates of early treatment and follow-up for this condition.

## Methods

### Ethical consideration

The study was approved by our institutional review board for research on human subjects at King Saud University, College of Medicine and Health Sciences, Riyadh, Saudi Arabia (approval E-18-3254 on May 31, 2018) and is in accordance with the National Committee of Bio Ethics Guidelines. Participants had full autonomy in answering survey questions with the ability to withdraw at any time. No rewards were offered to the participants. Moreover, written informed consent was obtained from all participants for publication of this study.

### Study design

This cross-sectional survey spanned 6 months, from June through November 2018, and was open to persons living in cities across all regions of Saudi Arabia. Potential participants included people of both genders and all age groups. Furthermore, the survey was only open to people living in Saudi Arabia who had not participated in any previous studies about clubfoot.

The minimal sample size, *n* = 308, was estimated with the following sample size equation (Cochran formula) based on a previous study [1] with the margin of error = 0.05, proportion of population with the attribute =72.3% [1] and Z score = 0.95 (Za = 0.95, *p* = 72.3%, d = 0.05):

$$ \left[{\mathbf{Z}}^{\mathbf{2}}p\left(\mathbf{1}-p\right)\right]/{\mathbf{d}}^{\mathbf{2}} $$

### Study instrument

A questionnaire to collect data on public awareness of clubfoot risk factors, treatment, and prognosis was designed by a group of orthopedic clinicians. Initial evaluation of the questionnaire was conducted in a pilot study with a sample of 80 participants who were not involved in the main study. Revisions to the questionnaire were made based on the pilot-test feedback. The final version was approved by consensus by all the authors, including a community medicine specialist.

The self-administered questionnaire was created using Google forms and distributed among the study participants. The questionnaire was made available to people in different shopping malls in several cities in Saudi Arabia where there were computer stations to complete the survey. At the beginning of the questionnaire, the participants were asked several questions regarding their sociodemographic data (age, gender, city of residence, educational level, and marital status). This section was followed by 11 main questions regarding clubfoot. If the respondent did not know about clubfoot, a simple introduction about the condition and a clinical figure illustrating the condition was provided. Participants were asked close-ended questions (Yes/ No) and multiple-option questions that assessed their awareness of, perceived causes of, and beliefs about clubfoot syndrome (Additional file 1).

### Statistical analysis

Categorical variables, such as gender and age groups, were expressed as frequencies and percentages. Multiple response dichotomy analysis was used to describe sources of information and perceived causes of clubfoot among newborns. Chi-square tests were used to assess the strength of association between demographic variables and knowledge about clubfoot. All analyses were performed with Statistical Package for Social Sciences software version 21 (SPSS, IBM, Armonk, NY, USA). The significance level was set to 0.05 for each analysis.

## Results

A total of 750 people completed the survey. Of these, 4 participants lived outside Saudi Arabia and were consequently not included in the data analyses. A summary of the participant demographic data is shown in Table 1.

**Table 1:** Demographic characteristics of respondents (*N* = 746)

Variables	Frequency (number)	Percent (%)
Sex		
Female	307	41.2
Male	439	58.8
Age group		
≤ 20 years	110	14.7
21–30 years	224	30
31–40 years	185	24.8
41–50 years	151	20.2
≥ 51 years	76	10.2
Marital status		
Single	284	38.1
Married	462	61.9
Educational level		
Secondary school or less	213	28.5
Diploma	25	3.4
Undergraduate degree	56	7.5
Higher studies	452	60.6
City size		
Small city	176	23.6
Large city	570	76.4

Table 2 summarizes the participant awareness of and perceptions about clubfoot. Most of the respondents (69.7%) had never heard or read about clubfoot. Of all the respondents, 5.4% had a child with clubfoot. The respondents were asked to select from a list of various options regarding their source of information about clubfoot to answer the remaining questions (Table 3). Our analysis showed that the top resource accessed by respondents was social media (38.4%, *n* = 87), followed by relatives and friends (19.9%, *n* = 45).

**Table 2:** Perceived causes, attitudes, and beliefs regarding clubfoot and its treatment (*N* = 742)

Questions and Responses	Frequency (number)	Percent (%)
Have you ever heard or read about clubfoot?		
No	520	69.7
Yes	226	30.3
Do you have a clubfooted child?		
No	706	94.6
Yes	40	5.4
^a^Are you informed about clubfoot because you have an affected child?		
No	5	12.5
Yes	35	87.5

^a^ Includes only the respondents who are parents of an affected child with clubfoot (*n* = 40)

**Table 3:** Summary of common sources of information on clubfoot

Variables	Frequency (*N* = 226^a^)	Percentage
Printed media (magazines, newspapers and books)	31	13.7
Websites	38	16.8
Television and radio	13	5.7
Relatives and friends	45	19.9
Social media	87	38.4
Affected persons	12	5.4

^a^ Includes only the respondents who were aware of clubfoot

Furthermore, the respondents were asked to select what they believed was the first line of treatment for clubfoot (cast, surgery, or physiotherapy) and when it was best to start treatment. A summary of responses is shown in Table 4.

**Table 4:** Summary of responses regarding knowledge of treatment for clubfoot^a^

Questions and Responses	Awareness of Clubfoot		*P*-value
	No	Yes	
To the best of your knowledge, what is the first line treatment of clubfoot?			
Cast	82 (15.8%)	65 (28.8%)	< 0.001
Physiotherapy	278 (53.5%)	110 (48.7%)	
Surgery	160 (30.8%)	51 (22.6%)	
To the best of your knowledge, when should clubfoot treatment be initiated?			
Do not know	7 (1.3%)	2 (0.9%)	0.001
Birth to the first 6 months	183 (35.7%)	95 (42.4%)	
First 6–12 months	156 (30.0%)	86 (37.1%)	
1–4 years	174 (33.4%)	43 (19.0%)	

^a^ Data are presented as frequency and percent unless otherwise specified

When asked about the efficacy of the various treatments, 30.2% of the respondents believed that between 41 and 60% of children born with a clubfoot improved after casting. On the other hand, 32.7% of the respondents thought surgery would improve the condition in 41–60% of the patients. About 33% of the respondents believed that physiotherapy was effective in 41–60% of clubfoot cases. The chi-squared test of association showed that females were significantly more aware of clubfoot (*p* <  0.001). However, the chi-squared test of independence showed that age, marital status, educational level, and city of residence did not correlate significantly with awareness of clubfoot. Conversely, people who had a child with a clubfoot were significantly more aware of the condition than those who had no child or relative with clubfoot (p <  0.001). A chi-square test of association suggested a statistically significant association between awareness and perception of first line treatment for clubfoot (p <  0.001). Respondents who believed cast placement was the first line of treatment for clubfoot were significantly more aware of clubfoot.

We also found that awareness of clubfoot was significantly associated with perception of the best time to initiate clubfoot treatment (*p* = 0.001). Respondents who believed that clubfoot treatment was best when the child was between 6 and 12 months old were significantly more likely to be aware of the condition. Similarly, a significant association was found between belief in the effectiveness of the cast as a treatment modality and awareness of the condition (*p* = 0.05). Participants who believed the cast was effective in 61–80% of cases were significantly more likely to be aware of clubfoot.

Awareness of clubfoot did not correlate significantly with beliefs in the effectiveness of surgery and physiotherapy for clubfoot. The chi-square test of independence showed that respondents who were aware of clubfoot had accessed significantly more printed media, websites, books, written media, and had learned more from relatives and affected persons than those who were unaware (*p* <  0.001 in each case). Also, a significantly low proportion of people among those who identified themselves as being aware of clubfoot suggested they learned about it from the current survey or identified themselves as having someone with this condition. Furthermore, the analysis showed that the perceived causes of clubfoot did not differ significantly between respondents who suggested they were aware of clubfoot and those who were unaware of the condition, except for those who believed fetal malposition (*p* < 0.001) or amniotic fluid deficiency (*p* = 0.020) were the causes of clubfoot (Table 5).

**Table 5:** Association between perception of causes of clubfoot and awareness of the condition

Perceived Causes of Clubfoot	Awareness of Clubfoot		*p*-value
	No = 520 N (%)	Yes = 226 N (%)	
Twin pregnancy	46 (8.8%)	26 (11.5%)	0.259
Sex of the newborn	11 (2.1%)	5 (2.2%)	0.933
“Evil eye” and witchcraft	25 (4.8%)	5 (2.2%)	0.097
Neurological disorders	212 (40.8%)	86 (38.1%)	0.486
Hereditary and genetic reasons	308 (59.2%)	128 (56.6%)	0.509
Mispositioned fetus	128 (24.6%)	87 (38.5%)	< 0.001
Cesarean section	40 (7.7%)	12 (5.3%)	0.240
Intrauterine deficient amniotic fluid	65 (12.5%)	43 (19%)	

## Discussion

We hypothesized that public awareness of clubfoot would be low since previous reports show that public knowledge varies from misconception to total ignorance [1–3]. In the current study, 520 (69.7%) of the respondents had never heard about clubfoot syndrome. Compared to other studies from other countries, the participants in the present study had a similar, low levels of awareness of clubfoot [1]. Alam et al. [2] assessed the knowledge of clubfoot in parents of clubfooted children and found that 93.3% of parents knew nothing about clubfoot before their children had the condition.

In Saudi Arabia, social media has become an important part of the everyday lifestyle. Approximately, 92% of the population uses the internet, and around 25 million are active users on social media [7]. Thus, it was expected that social media would be selected as the most common resource for learning about clubfoot (38.4%) by our respondents who knew about clubfoot. In addition, 19.9% of the respondents learned about clubfoot from a relative or friend, whereas only 16.8% had learned about the condition via a health website. While we found that only 5.7% of our respondents learned about clubfoot through watching television or listening to the radio. These results indicate that social media might be an effective option for an awareness campaign. Further studies are needed to determine the effectiveness of different awareness campaign models and how they in turn effect patient outcomes.

From previous studies, the most widely accepted theory among orthopedic specialists is that clubfoot is caused by a combination of genetic and environmental factors [4, 8]. Additionally, having a parent with clubfoot may increase the chances of having a child with the condition [3]. A neuromuscular etiology has been proposed based on histochemical analysis of muscle specimens from people with clubfoot [8]. In addition, an alternative theory of arrested fetal development has been proposed. It has been reported that clubfoot is inherited as a polygenic multifactorial trait, which implies that genetic factors do play an important role in the development of this condition [8]. In some communities, astronomical events are believed to be responsible for clubfoot [3]. In our survey, the most prevalent perceived cause of clubfeet was hereditary and genetic disorders (58.4%), followed by neurological disorders (39.9%). Therefore, it is important that the public receives correct knowledge about the etiology so that parents can seek proper treatment from orthopedic specialists.

The best initial treatment of clubfoot is non-surgical, regardless of the severity of the deformity. The Ponseti method (or casting method) for clubfoot correction is the most frequent method used worldwide. This method uses gentle stretching and casting to gradually correct the deformity [5]. In our survey, only 19.7% of the respondents were aware that casting was the best initial treatment for clubfoot. Approximately 52.0 and 28.3% of the respondents thought physiotherapy and surgery were the first line of treatment, respectively. In a study conducted by Burfat et al. [3] most participants thought that surgery was the most common treatment modality. Additionally, participants thought that there were several traditional methods for the treatment of clubfoot, with the most frequent being oil massages or warm bandages. These results show that most of the population knew little about the cast technique, perhaps not understanding how a deformed foot could be corrected by cast only. Awareness regarding clubfoot among the general population may improve patient outcomes due to early treatment and more consistent follow-up. Anuar et al. [9] concluded in their study that developmental hip dysplasia should be detected as early as possible in neonates in order to improve patient outcomes. They recommended that neonatal screening programs must include strategies to raise awareness among the parents and should not be limited to evaluation of the infant in order to decrease the number of late-presenting hip dysplasia cases. The authors posit that this will subsequently lessen the economic burden on the government. Therefore, we suggest implementing a similar method for clubfoot. Ideally, further studies will analyze the effectiveness of such interventions on patient outcomes.

Rasheed et al. [1] reported that only 14 respondents (11.89%) in their survey were knowledgeable about the Ponseti technique (casting method), whereas the remainder of the respondents did not know about the technique. Also, when asked about the best treatment methods for clubfoot (surgery or the Ponseti technique), 90 (57.6%) of the respondents selected the Ponseti technique compared to 23 (19.3%), who selected surgery for clubfoot management [1].

Our analyses showed that 1.2% of the respondents did not know the best time to start treatment for clubfoot. 37.7% of the participants responded that treatment should be offered before the age of 6 months, whereas 32.8% thought it was best to start treatment between 6 and 12 months. Alam et al. [2] reported that 11.8% of the participants in their survey did not know the best time to begin the treatment of clubfoot, and 88.2% said that the best time for treatment was immediately after birth.

Although the public is not expected to know the method or the technique of the treatment for clubfoot in detail, similar findings regarding treatment modality were concluded from another study and were useful for assessing whether the population thinks that this condition can be treated by cast rather than surgery [3]. This factor is an important part of awareness since there are many neglected or late-presenting cases due to several barriers to treatment. One is family perception that the condition must be treated surgically. This may raise parents’ fears about the use of general anesthesia during a crucial time of brain development. Therefore, misinformation regarding the need for surgery may delay consultation and treatment. Another misperception may be that the condition can be corrected by itself or with the aid of a special boot. Much of this misinformation is spread over social media which is a major resource for many parents. In our survey, 30.2% of the respondents believed that between 41 and 60% of clubfooted children improved after casting, as opposed to 32.7% who thought surgery would improve the condition in 41–60% of the patients. Bridgens et al. [8] conducted a review to compare the outcomes of surgery and casting (Ponseti) for clubfoot and found that 43% of the cases treated surgically had an excellent or good result compared with 78% who were treated using the Ponseti method. In our survey, about one-third of the respondents believed that physiotherapy was effective in 41–60% of patients, whereas a smaller proportion (25.3%) believed physiotherapy was effective in 61–80% of children with clubfoot. These results indicate a lack of knowledge about clubfoot treatment in the general population. Thus, it is necessary to increase the awareness of the population about the disease since early treatment is associated with better outcomes. Awareness campaigns on a national level may improve early treatment and follow-up rates. Results from this study can be used for guiding strategy, such as the recommendation to use social media as a primary platform for a national awareness campaign, as well as serving as baseline data for evaluating these types of initiatives.

### Limitations

The limitations of this study warrant consideration. First, most of the participants who completed the survey were from major cities in Saudi Arabia. Therefore, more data from the countryside or small regions are needed for optimizing the strength of the results. Second, the lack of similar studies in the larger Arabian Peninsula makes it difficult for us to make relevant comparisons. Third, due to the small sample size of this study, it is difficult to generalize the findings. Thus, we recommend conducting larger studies with aid of the government to obtain better results.

## Conclusion

Within the limitations of the present study, that the results suggest that lack of public knowledge regarding clubfoot may be a barrier to early intervention and successful management of this condition. Social media holds promise as a tool to increase public awareness, but only in collaboration with reliable sources, such as healthcare providers.

## Supplementary information

**Additional file 1.** Questionnaire to assess public knowledge of clubfoot.

## Data Availability

The datasets used and analyzed during the current study are available from the corresponding author on reasonable request.
