# Supplementary material for: Assessing public awareness of clubfoot and knowledge about the importance of early childhood treatment: a cross-sectional survey
Source: BMC Pediatr. 2019 Oct 17;19:358. doi: 10.1186/s12887-019-1740-z (PMC6796333; doi:10.1186/s12887-019-1740-z)
Supplement: Supplementary file 1 — Additional file 1. Questionnaire to assess public knowledge of clubfoot. [file 12887_2019_1740_MOESM1_ESM.docx]

**Additional file 1. Questionnaire to assess public knowledge of clubfoot**

1. Have you ever read or heard about clubfoot?

2. Which of the following resources have you consulted?

3. Do you have a child with clubfoot?

4. Is it because you have a child with clubfoot that you know about the disease?

5. Which of the following factors do you think increases the risk for clubfoot?

6. What is the first treatment for clubfoot?

7. What is the proper age to treat clubfoot?

8. What do you think is the percentage of improvement if treated by physiotherapy?

9. What do you think is the percentage of improvement if treated by serial casting?

10. What do you think is the percentage of improvement if treated by surgery?

11. In your opinion, what proportion of clubfoot patients need surgical treatment?
